# A latent profile analysis of opioid-related knowledge, social relationships, and campus connectedness among college students

**DOI:** 10.3389/fpubh.2026.1844206

**Published:** 2026-06-23

**Authors:** Christina E. Freibott, Michael D. Stein, Sarah Ketchen Lipson, Noel Vest

**Affiliations:** 1 Department of Community Health Sciences, Boston University School of Public Health, Boston, MA, United States; 2 Department of Health Law, Policy, and Management, Boston University School of Public Health, Boston, MA, United States

**Keywords:** campus connectedness, college students, latent profile analysis, naloxone, opioid overdose

## Abstract

**Objectives:**

To identify distinct patterns of opioid-related knowledge, social relationships, and campus connectedness in a national sample of college students.

**Methods:**

Data were collected from 7,087 students (ages of 18–25) from 17 colleges and universities who participated in the 2021–2022 Healthy Minds Study (HMS).

**Results:**

Using latent profile analysis (LPA), four profiles were empirically derived: Profile (1) high knowledge and willing to intervene during an overdose (14%); Profile (2) disconnected from campus and resources (12%); Profile (3) neutral/hesitant (14%); and Profile (4) low knowledge but willing to intervene during an overdose (60%). There were significant differences in opioid misuse, naloxone knowledge, presence of school-based Good Samaritan policies, and mental health status between profiles.

**Conclusions:**

While opioid-related knowledge was generally low, the majority of students felt connected to their campus community and were willing to call for help during an overdose event. By understanding what proportion of their student body falls into each profile, schools can tailor their approach. These results can inform tailored efforts at colleges and universities to implement overdose education programs, expand naloxone access, or implement Good Samaritan policies on campus.

## 1 Introduction

The rate of unintentional drug overdose deaths among adolescents and young adults (AYAs) increased 147% between 2015 and 2022, attributed in large part to the proliferation of fentanyl in the drug supply (1). A growing number of colleges and universities are providing opioid overdose education and expanding access to naloxone (used to reverse an opioid overdose) to prevent overdose fatalities at their institutions (2, 3). Many schools have also established Good Samaritan or medical amnesty (GSMA) policies, which protect students from disciplinary action when calling for help during a drug or alcohol-related incident. These policies encourage bystander intervention among college peers, who are most likely to be present in settings where AYAs are using substances. A national report found that approximately one-third of AYAs knew someone who experienced an opioid overdose, indicating that college peers may be uniquely positioned to intervene with life-saving measures (4).

Prior research demonstrates that relationships within communities or social networks have implications for health-related behaviors, particularly for AYAs (5–7). For example, perceptions of peer substance use and social norms can impact an individual's substance use behaviors (8, 9). A study examining misperceptions of prescription drug misuse among college students found that 75% of the study sample thought their peers used prescription painkillers in the past month, in contrast to the 5% who reported actual past month use (9). These misconceptions can normalize high risk substance use within AYA populations (9). Relatedly, rates of mental health service utilization nearly doubled among college AYAs between 2007 and 2017, which may be due to decreased stigma among peers regarding mental health treatment (10). A recent social norms intervention to improve mental health-related attitudes and behaviors among college students found that students with more positive perceptions of campus norms toward mental illness had lower levels of stigma (11). Due to the co-occurrence of mental health and substance use behaviors, programs addressing peer perceptions and norms for both mental health and substance use may significantly influence AYAs (12).

Given the importance of peers as trusted sources of information and influence, and the rise in opioid overdoses, there is a need to better understand how AYAs may react during an overdose scenario (7). While two-thirds of opioid-overdose fatalities from 2019 to 2021 among adolescents had one or more bystanders present, there was no bystander response in nearly 70% of those overdose deaths (13). A number of programs and interventions leverage the role of peers, particularly in the higher education setting—as there are more than 20 million AYAs enrolled in colleges and universities across the US (14). Many interventions emphasize teaching students to be prosocial bystanders, providing education to improve knowledge and overdose assistance self-efficacy to increase the likelihood of intervening during a difficult scenario (15).

A recent study reported that the majority of college AYAs are willing to call for help during an overdose scenario, but few know how to administer naloxone (16). Further, students who received training with naloxone were found to have greater knowledge of overdose risk, signs, and bystander actions than those without training (17). While these studies provide a link between naloxone training and opioid-related knowledge, they utilize variable-based approaches (e.g., focusing on associations with a single outcome) and do not consider social and community factors. This is important, as the college setting can vary significantly between institutional contexts (e.g., urban/rural, public/private, student body size), and student knowledge and behavior may also differ between these settings. A person-centered approach (e.g., categorizing college students into subgroups with similar patterns of opioid-related knowledge) along with the examination of social relationships and campus connectedness may provide a more useful characterization of willingness to intervene during an overdose among college AYAs.

Campus connectedness, defined as a student's sense of belonging to and fitting in with their university community, is particularly salient during young adulthood, when identity and interpersonal bonds are evolving (18–21). Self-categorization theory provides a valuable framework for understanding how these social and cognitive factors interact to influence intervention behaviors. This theory posits that individuals derive a social identity from groups with which they feel connected, adopting group goals and motives as their own and acting on behalf of the group when that identity is salient (22, 23). Applying this to a campus context, students who feel a strong connection to their university may be more motivated to protect fellow students and promote campus safety, thereby increasing their likelihood to intervene in critical situations, such as opioid overdose events (49, 50). By exploring opioid-related knowledge, social relationships, and campus connectedness through latent profile analysis (LPA), this study seeks to identify subgroups of students who are most likely to act effectively in overdose scenarios.

## 2 Materials and methods

### 2.1 Sample and procedures

Data come from the 2021–2022 *Healthy Minds Study* (HMS), a web-based survey administered annually to random samples of college students at higher education institutions across the US (24). Details about the study design of HMS have been documented extensively in prior publications (24–27). HMS includes three required modules (demographics, mental health status, mental health service utilization/help-seeking) and 15 elective modules. Students in this sample (*n* = 11,233) were those whose institution opted-in to the substance use elective module. Institutional review boards at all schools approved the survey. Students who were 18 or older were recruited via email, and upon clicking a personalized link in the recruitment email, were presented with informed consent before completing the survey. Participation incentives in the form of eligibility for one of 12 cash prizes (two $500 and 10 $100 gift cards) were provided. Data were collected using Qualtrics software (28). At colleges and universities with more than 4,000 students, a random sample of 4,000 degree-seeking students were invited to participate. At smaller institutions, all students were invited to participate.

### 2.2 Demographic characteristics

Student-level characteristics included in this model are gender, sexual orientation, race/ethnicity, undergraduate year, degree, extracurricular participation (e.g., Greek life, athletics), substance use, living place, and depression or anxiety symptoms. Institutional-level characteristics included in this model are student body size, type (e.g., Associate's College, Doctoral Universities), geographic region, and public/private designation.

### 2.3 Indicator variables

Latent profile analysis (LPA) is a person-centered approach that quantitatively identifies subgroups (latent profiles) within a sample. LPA uses participant responses to indicator variables (e.g., variables assessing opioid-related knowledge or peer relationships) to identify patterns in responses across all questions and organize them into groups based on their response patterns (29, 30). There were eight indicator variables included in this analysis, which are described below.

#### 2.3.1 Opioid-related knowledge

Four questions assessing opioid-related knowledge were included as indicator variables. These were adapted from the Opioid Overdose Knowledge Scale (31) and Opioid Overdose Attitudes Scale (31), and targeted college student populations. The questions were:

1. **Overdose Recognition:**“To the best of your knowledge, which of the following are indicators of an opioid overdose?” (Select all that apply from a list of 10 symptoms, 6 of which were correct. Scores for this variable include the number of correct responses.).
2. **Naloxone Use:** “I know how to use naloxone if someone overdoses.” (Responses: completely disagree, disagree, unsure, agree, completely agree).
3. **Willingness to Intervene:** “I would be concerned about calling emergency services in case I get into trouble with my school or the police come.” (Responses: completely disagree, disagree, unsure, agree, completely agree).
4. **Prescription Drug Risk:** “How much do you think people risk harming themselves if they use prescription drugs not prescribed to them?” (Responses: no risk, slight risk, moderate risk, great risk).

#### 2.3.2 Social and campus connectedness

Two questions pertaining to social relationships were included as indicator variables. Both questions come from the *Flourishing Scale*, which is an 8-item survey assessing self-perceived success in regards to personal relationships, self-esteem, purpose, and optimism (32).

1. **Rewarding Relationships:** “My social relationships are supportive and rewarding.” (Responses: strongly disagree, disagree, slightly disagree, mixed or neither agree nor disagree, slightly agree, agree, strongly agree).
2. **Positive Relationships:** “I actively contribute to the happiness and well-being of others.” (Responses: strongly disagree, disagree, slightly disagree, mixed or neither agree nor disagree, slightly agree, agree strongly agree).

Two questions pertaining to campus connectedness were included as indicator variables.

1. **Campus belonging**: “I see myself as part of the campus community.” (Response options strongly agree, agree, somewhat agree, somewhat disagree, disagree, strongly disagree).
2. **Campus resources**: “If I needed to seek professional help for my mental or emotional health, I would know where to access resources from my school.” (Response options strongly agree, agree, somewhat agree, somewhat disagree, disagree, strongly disagree).

#### 2.3.3 Predictors of profile membership

Predictors of latent profile membership included two domains: naloxone knowledge and institution-level opioid policies.

1. **Naloxone knowledge:** “To the best of your knowledge, what is naloxone used for?” (Response coded to correct/incorrect).
2. **Institution-level opioid policies:** Presence/absence of policies were determined from a college-level policy tracking database, which include Good Samaritan policies, medical amnesty policies, and opioid overdose prevention programs.

#### 2.3.4 Concurrent outcomes

Concurrent outcome variables included two domains: student-reported perceptions of peer substance use and mental health symptoms.

1. **Perceptions of peer substance use:** “In the past 30 days, about what percent of students at your school (drank alcohol; smoked cigarettes; smoked or used marijuana; vaped).”
2. **Mental health symptoms:**

a Depression symptoms were calculated with responses to the Patient Health Questionnaire (PHQ-9) (33), and a score of 10 or greater constitutes a positive screen.
b Anxiety symptoms were calculated with responses to the Generalized Anxiety Disorder 7 (GAD-7) (34), where a positive screen is a score of 10 or greater.
c Loneliness was measured using the UCLA 3-item loneliness scale (35), which measures relational connectedness, social connectedness, and self-perceived isolation, with a score of 6 or greater indicating the respondent is “lonely.”

### 2.4 Analysis

**Figure 1:**
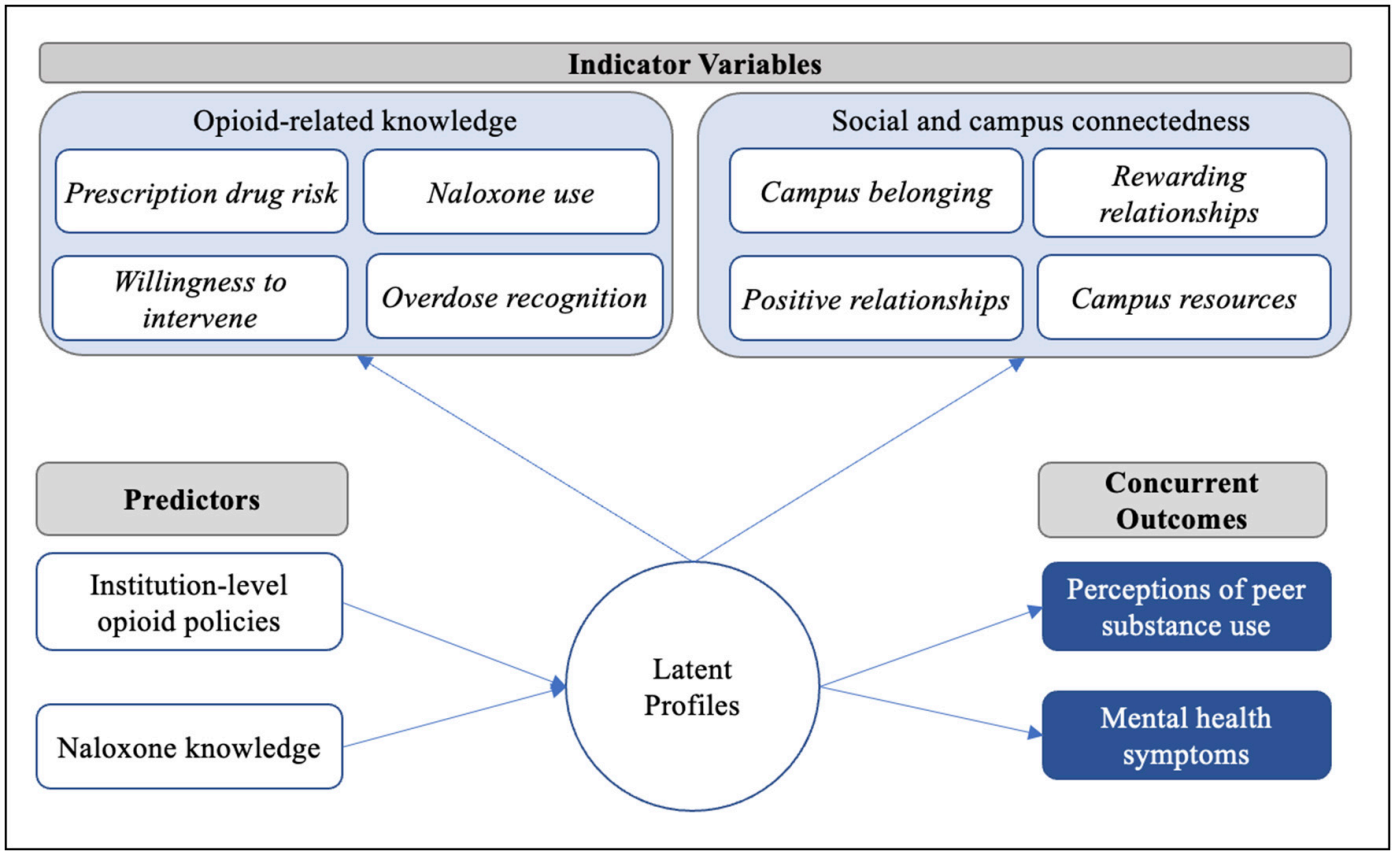
Diagram of the full latent profile model with included predictors.

Using administrative data (i.e., including sex, race/ethnicity, and GPA) from each institution, sample probability weights were constructed by the *HMS* team and assigned to each participant, so that those less likely to respond to the survey were given a larger weight (36). Our primary outcome was a latent profile analysis (LPA) of college students based on their opioid-related knowledge and levels social and campus connectedness (see Figure 1). We entered into the model all eight indicator variables.

The optimal number of latent profiles is determined using the following model fit indices: Akaike Information Criterion (AIC), Bayesian Information Criterion (BIC), Lo-Mendell-Rubin adjusted likelihood ratio test (LRT), entropy values, and profile size (37). AIC and BIC assess model fit with lower values indicating better fit, LMR-LRT compares current model with a model having one less class (*k* – 1), and entropy measures classification accuracy, with ≥0.8 indicating adequate precision (30, 38). The LPA was calculated using Mplus version 8.10 (39). After selecting the optimal number of latent profiles, *Z*-scores, or standardized scores demonstrating the scores value to the mean, were calculated for each indicator to simplify comparison across indicators and between latent profiles.

We assessed differences between profiles for concurrent outcomes in Mplus using the BCH approach (equality tests of means across classes with 3 degrees of freedom for the overall test), while the R3step method was used for predictors of profile membership (40, 41). The BCH and R3step methods are preferred because they account for classification error in latent profile assignment, preserving the integrity of the latent profile solution when examining distal outcomes and predictors. These methods reduce bias and produce more accurate estimates by appropriately modeling the uncertainty in class membership (30). Descriptive statistics for demographic characteristics were computed using Stata/MP 17.0 (42).

## 3 Results

### 3.1 Participant characteristics

Our sample included 7,087 students between the ages of 18 and 25 from 17 colleges and universities. Of the included participants, 67% were female, 67% were heterosexual, and 65% were white. The average age was 20.6 (*SD* 2.0) with 32% of respondents in their first year of college.

### 3.2 Model selection

The two to five profile models were tested to determine the most parsimonious model (Table 1). While the five-profile solution has a highest entropy and non-significant LRT *p*-value, it also created a profile with < 5% of the sample. Generally, profiles with a small proportion of the students (< 5%) are considered not clinically relevant, as it would not be as effective to create an intervention for those in the vast minority (43). Therefore, we identified the 4-profile solution as the most parsimonious fit to the data as well as the most clinically relevant.

**Table 1:** Fit indices for the latent profile analysis.

Number of Profiles	AIC	BIC	SABIC	Profile size %	LRT *p*-value	Entropy
2 profiles	182,201	182,372	182,293	81, 19	< 0.001	0.841
3 profiles	179,380	179,614	179,506	60, 13, 27	< 0.001	0.879
4 profiles	**177,433**	**177,728**	**177,592**	**14, 12, 14, 60**	**< 0.001**	**0.882**
5 profiles	176,549	176,906	176,741	13, 3, 59, 12, 13	0.002	0.894

AIC, Akaike information criteria; BIC, Bayesian information criteria; SABIC, sample size adjusted Bayesian information criteria; LRT, Lo-Mendell-Rubin adjusted likelihood ratio test. Bolded values denote that the model was selected as the most parsimonious.

### 3.3 Description of latent profiles

Profile one: **High knowledge and willing to intervene** (14% of sample) profile was characterized by students knowing how to use naloxone, being able to identify at least three signs of an opioid overdose, and not being concerned about calling emergency services during an overdose event.

Profile two: **Disconnected from campus and resources** (12%) profile was characterized by students who do not feel part of the campus community and peers, do not know where to seek help for mental or emotional health, and do not know how to use naloxone.

Profile three: Neutral or hesitant (14%) profile was characterized by students who were the most concerned about calling emergency services during an overdose event, and feel connected to their campus and peers.

**Figure 2:**
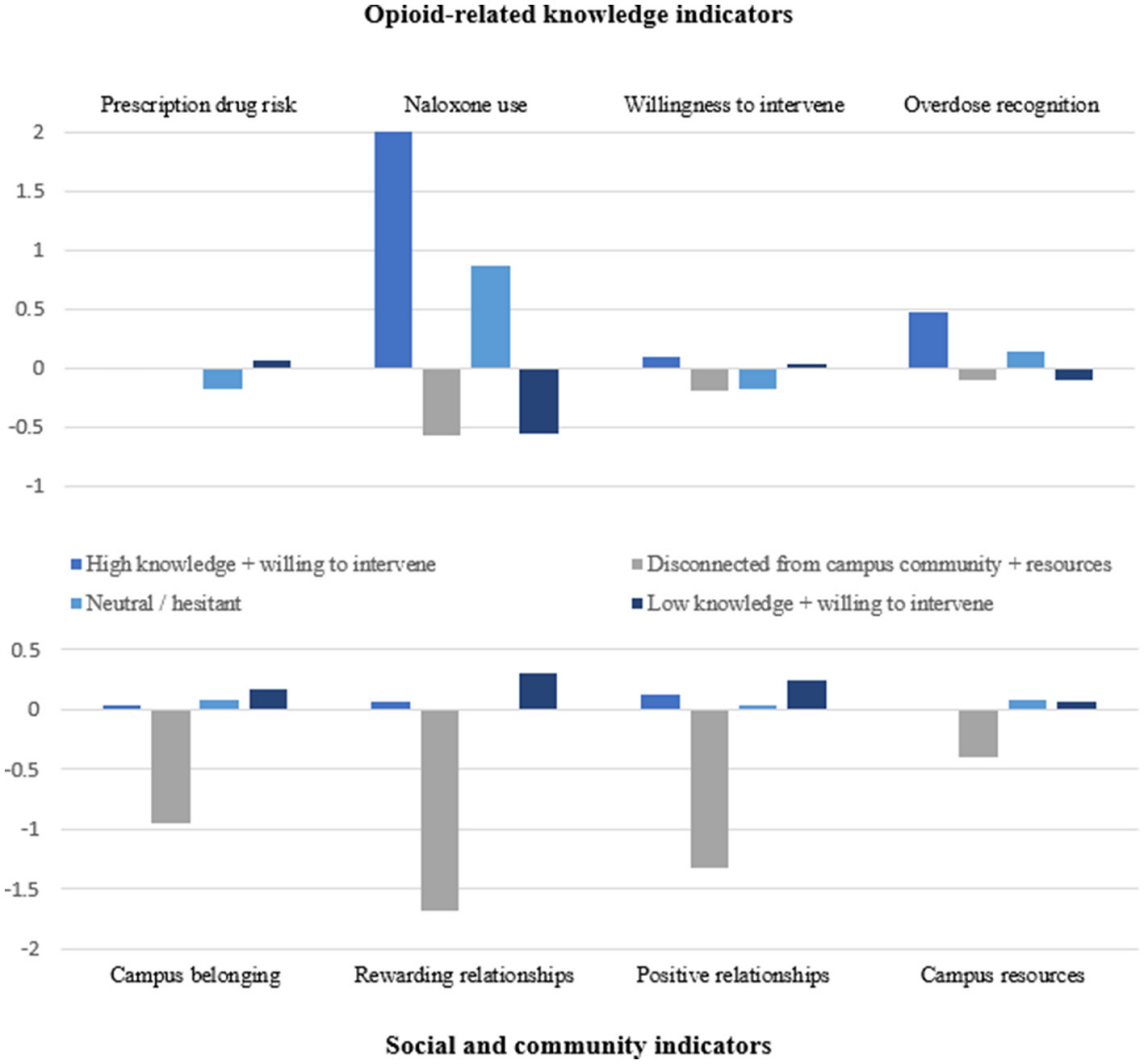
Indicator variables by latent profiles (*z*-scores).

Profile four: **Low knowledge but willing to intervene** (60%) profile was characterized by students who felt connected to their campus and peers, who had the lowest levels of naloxone and opioid-related knowledge, but were among the most willing to call emergency services (second to the High knowledge group). Figure 2 depicts differences in responses to indicator variables, stratified by latent profiles. Complete student- and institutional-level descriptives, stratified by latent profile, are reported in Table 2.

**Table 2:** Student- and institutional-level demographics stratified by latent profile (%).

	Profile 1	Profile 2	Profile 3	Profile 4	Total
	*n* =1,006	*n* = 870	*n* = 963	*n* = 4,248	*n* = 7,087
Student demographics					
**Age** ^*^	20.8 (0.1)	20.5 (0.1)	20.5 (0.1)	20.6 (0.1)	20.6 (0.1)
Year in college					
1	27.7	33.5	34.4	32.3	31.8
2	24.9	27.0	27.0	26.4	26.1
3	22.3	21.5	22.3	22.4	22.1
4	20.3	14.8	14.5	16.6	16.5
5+	4.7	3.3	1.8	2.3	2.7
Gender					
Male	25.4	32.0	27.2	27.3	27.1
Female	69.2	59.9	66.2	67.7	65.7
TGNC	5.4	8.0	6.6	5.0	5.6
Race and ethnicity					
White	70.0	63.8	59.9	64.9	64.7
Asian	11.4	11.9	17.3	14.1	13.9
Latinx	7.9	11.1	10.9	9.9	9.9
Black	6.4	8.7	7.8	7.3	7.4
Middle Eastern	1.7	1.6	1.9	1.8	1.7
AN/PI	1.3	1.4	1.0	1.2	1.2
Other	1.3	1.6	1.2	0.9	1.1
Sexual orientation					
Heterosexual	68.8	58.0	65.1	69.4	66.7
LGBQ	31.2	42.0	34.9	30.6	32.3
Living place					
Residence hall	30.2	37.1	35.1	36.9	35.7
On-campus apartment	9.9	11.4	9.0	12.4	11.4
Fraternity or sorority	0.6	0.7	0.8	0.7	0.7
Co-operative housing	0.4	1.3	1.2	0.6	0.7
Off-campus housing	42.5	28.0	35.1	33.4	34.2
Parents	16.0	20.7	17.5	15.2	16.3
Other	0.5	0.9	1.1	0.8	0.8
Lifetime diagnoses					
Depression	29.0	37.8	27.3	23.6	52.4
Anxiety	39.6	39.2	33.8	31.3	73.4
**Past-month opioid misuse**	1.4	0.7	0.4	0.3	0.5
Institutional characteristics Size					
< 5,000	18.4	16.5	20.6	18.3	18.4
5,000-19,999	35.0	35.7	31.6	33.6	33.8
>20,000	46.6	47.7	47.8	48.0	47.7
Type					
Community colleges	4.2	5.3	5.9	4.0	4.5
Four-year colleges and universities	90.2	86.2	85.5	89.2	88.4
Special focus institutions	5.7	8.5	8.5	6.8	7.1
Geography					
New England	35.0	37.1	37.9	35.7	36.1
Mid-Atlantic	4.9	4.6	3.9	3.9	4.1
South Atlantic	14.0	12.1	15.5	13.7	13.8
East South Central	38.3	35.5	34.8	34.9	35.5
West South Central	3.1	4.8	3.4	3.4	3.5
West	4.8	5.9	4.5	8.3	7.0

^*^Age reported as mean age in years with the standard error in parentheses e.g., “mean (SE)”.

### 3.4 Predictors of profile membership

Complete results are reported in Table 3. 30.2% of students correctly identified that naloxone is used to reverse the effects of an opioid overdose, 64.6% attended institutions with a Good Samaritan or medical amnesty (GSMA) policies, and 45.1% of students attended colleges or universities with opioid overdose prevention programs. There was a statistically significant difference in opioid misuse (χ^2^ = 19.146, *p* < 0.001), naloxone knowledge (χ^2^ = 496.739, *p* < 0.001), and presence of GSMA policies (χ^2^ = 15.29, *p* = 0.002) between latent profiles. There were not statistically significant differences in the presence of opioid prevention programs across the profiles.

**Table 3:** Overall profile membership totals and odds ratios with 95% confidence intervals for baseline predictors.

% odds ratio (95% *CI*)	High knowledge + willing to intervene	Disconnected from campus and resources	Neutral/hesitant	Low knowledge + willing to intervene (referent)
School policies				
GSMA	**60.2%**	66.1%	**62.1%**	66.0%
	**0.78 (0.7, 0.9)**	1.00 (0.8, 1.2)	**0.848 (0.7, 0.9)**	
Overdose prevention	45.9%	45.6%	43.2%	45.2%
	1.03 (0.9, 1.2)	1.02 (0.9, 1.2)	0.92 (0.8, 1.1)	
Naloxone knowledge	**56.3%**	21.6%	**40.7%**	23.5%
	**4.41 (3.8, 5.1)**	0.90 (0.7, 1.1)	**2.32 (2.0, 2.7)**	

Odds ratios were computed using the R3STEP approach in the Mplus software package. Bolded values indicate that the profile is significantly different when compared to the referent profile (low knowledge + willing to intervene) at the *p* < 0.05 level.

### 3.5 Concurrent outcomes

On average, students estimated that in the last 30 days: 69% drank alcohol, 28% smoked cigarettes, 45% smoked or used marijuana, and 55% vaped. There were no significant differences in peer perceptions of substance use between latent profiles.

The average PHQ-9 depression score across all latent profiles was 9.7 (95% *CI* 9.5–9.9), average GAD-7 anxiety score was 8.5 (95% *CI* 8.4–8.6), and the average UCLA 3-item loneliness score was 5.9 (95% *CI* 5.9–6.0). There was a statistically significant difference in depression (χ^2^ = 439.042, *p* < 0.001), anxiety (χ^2^ = 215.363, *p* < 0.001), and loneliness symptoms (χ^2^ = 1,034.564, *p* < 0.001) across latent profiles. As compared to the Low knowledge but willing to intervene class, all other profiles had significantly higher scores (e.g., higher levels of symptoms) on mental health scales. Complete results are reported in Table 4.

**Table 4:** Overall profile membership means and chi-square for concurrent outcomes.

Mean (*SE*)	High knowledge + willing to intervene	Disconnected from campus and resources	Neutral/hesitant	Low knowledge + willing to intervene (referent)	Chi-square (*p*-value)
Peer substance use					
Alcohol	67.4 (2.3)	68.9 (2.5)	73.2 (2.1)	68.4 (1.1)	4.4 (0.2)
Cigarettes	28.5 (2.2)	25.5 (2.7)	29.5 (2.0)	27.5 (1.1)	1.6 (0.6)
Cannabis	48.0 (2.5)	47.7 (3.0)	47.0 (2.4)	43.0 (1.2)	5.2 (0.1)
Vaped	56.7 (3.0)	55.7 (3.9)	53.6 (2.9)	54.3 (1.3)	0.8 (0.9)
Mental health					
Depression	**9.7 (0.2)**	**14.9 (0.3)**	**9.8 (0.2)**	8.5 (0.1)	439.0 (< 0.001)
Anxiety	**8.8 (0.2)**	**11.7 (0.2)**	**8.6 (0.2)**	7.7 (0.1)	215.3 (< 0.001)
Loneliness	**5.9 (0.6)**	**8.0 (0.1)**	**5.8 (0.1)**	5.6 (0.1)	1,034.6 (< 0.001)

Chi-square and *p*-values were computed using the BCH approach in the Mplus software package with 3 degrees of freedom. Bolded values indicate that the profile is significantly different when compared to the referent profile (low knowledge + willing to intervene) at the *p* < 0.001 level. Depression was measured using the PHQ-9, for which a score of 10 or greater indicates a positive screen for depression. Anxiety was measured using the GAD-7, for which a score of 10 or greater indicates a positive screen for anxiety. Loneliness was measured using the UCLA 3-item loneliness scale, for which a score of 6 or greater indicates the respondent is lonely.

## 4 Discussion

This is the first study to use a national sample of college students to characterize willingness to intervene during an opioid overdose. The largest group (60%) was the low knowledge but willing to intervene profile, while the remaining three profiles—high knowledge + willing to intervene, neutral or hesitant, disconnected from campus and community resources—were of similar sizes (14%, 14%, 12%, respectively). Our findings suggest that while opioid-related knowledge is generally low, the majority of students feel connected to their campus community and are willing to call for help during an overdose event.

We found no significant differences between latent profiles with regard to perceptions of peer use of alcohol, cigarettes, cannabis, and vaping. This suggests that our sample was generally in agreement about how much they think their peers use different substances. Prior research has demonstrated these beliefs about peer substance use may not be accurate, and that social norms campaigns may be important in correcting these false assumptions (9). There was also general agreement with regard to prescription drug risk, with the majority of students indicating that there is moderate to great risk in someone using prescription drugs not prescribed to them. Current research indicates that increases in overdose deaths among AYAs are largely due to the presence of fentanyl in counterfeit pills (44). As approximately 1 in 10 college students have taken a pill without knowing its formulation, this indicates an area for more widespread education or intervention efforts among college students (4).

When assessing mental health characteristics, we found that there were significant differences in depression, anxiety, and loneliness symptoms for all profiles (high knowledge + willing to intervene, disconnected from campus & community, neutral/hesitant) when compared to the low knowledge but willing to intervene profile. The disconnected from campus & resources class had the highest depression (14.9), anxiety (11.7), and loneliness (8.0) scores, scores indicating positive screens for clinically significant symptoms. Notably, this class also had high baseline levels of lifetime depression (37.8%) or anxiety (39.2%) diagnoses, indicating a consistent need for mental health support that may predate their transition to college. Further, this class had the lowest levels of knowledge of resources for mental or emotional health, which suggests that campus-based resources may not be reaching some students with high levels of need.

The majority of students in our sample had positive social relationships and considered themselves part of their campus community. However, a small but consistent minority of students in the disconnected from campus community + resources profile did not. Importantly, this profile had the highest percentage of LGBQ (42.0%) and TGNC (8.0%) students. Prior research has shown that, depending on institutional context, LGBTQ+ students may feel less welcome than their cisgender or heterosexual peers. For example, LGBTQ+ who have positive perceptions of their campus diversity climate were more likely to feel a sense of belonging (45). This may also be affected by state-level policies that can positively or negatively affect the types of resources colleges and universities can have for LGBTQ+ students (46).

The majority of schools included in our study were 4-year colleges and universities of at least 5,000 students. Approximately two-thirds of students attended schools with a GSMA policy. There were significant differences in presence/absence of GSMA policies between latent profiles: the high knowledge + willing to intervene and neutral/hesitant classes were less likely to be at a campus with a GSMA policy. It may be that, regardless of school policy, there is a core group of students who have high levels of opioid-related knowledge and would intervene during an overdose, regardless of GSMA policy or potential disciplinary ramifications. While GSMA policies have been reported to increase help-seeking during alcohol-related emergencies, these policies are poorly understood in the context of help-seeking during opioid overdose events (47, 48). Future research should explore if students are aware of their institution's GSMA policy and if it influences their behavior during an overdose event.

Although this study is the first to assess latent profiles of opioid-related knowledge in a large, multi-campus sample of college students, it is not without limitations. First, these data are self-reported, and participants may have under or over-estimated their opioid-related knowledge, quality of their social relationships, and/or feelings of community connectedness. However, LPA allows us to look at patterns of responses across many questions, which minimizes concern with self-report bias to an individual question. Second, there may be other factors associated with opioid-related knowledge that influence student behavior beyond social relationships and campus connectedness. Future research should explore how (and if) these latent profiles are altered when considering additional indicator variables. Third, we operationalize willingness to intervene through responses to the question asking if students are concerned about calling emergency services for fear of getting into trouble with their school or law enforcement. This represents just one aspect of intervening (e.g., calling for help), and future research should explore additional intervention behaviors (e.g., administering naloxone). Further, we do not know how many in our sample have received opioid overdose education, administered naloxone, or witnessed an overdose. Lastly, as these data are cross-sectional, we cannot make inferences about how these latent profiles may change over time. While we report results from a multi-institutional study, even more large-scale research with dozens of schools is needed moving forward.

## 5 Conclusion

This is the first study to use a person-centered approach to identify profiles of college students and their willingness to intervene during an overdose scenario. Our results indicate that the majority of students are willing to intervene during an overdose scenario, despite low levels of opioid-related knowledge. These results can guide intervention planning for colleges and universities who are seeking to implement, or augment, substance use education and prevention programs. By understanding what proportion of their student body falls into each profile, schools can tailor their programs to be primarily education based (low knowledge but willing to intervene profile) or improve their outreach and marketing strategies to students who may not be aware of existing campus resources (disconnected from campus and community resources profile). Future research should also explore how school-level policies may affect students' knowledge or willingness to intervene during an overdose scenario, as fear of disciplinary consequences may inhibit action regardless of resources available.

## Data Availability

Publicly available datasets were analyzed in this study. This data can be found here: https://healthymindsnetwork.org/research/data-for-researchers/.
